# Observation of acceleration and deceleration in gigaelectron-volt-per-metre gradient dielectric wakefield accelerators

**DOI:** 10.1038/ncomms12763

**Published:** 2016-09-14

**Authors:** B. D. O'Shea, G. Andonian, S. K. Barber, K. L. Fitzmorris, S. Hakimi, J. Harrison, P. D. Hoang, M. J. Hogan, B. Naranjo, O. B. Williams, V. Yakimenko, J. B. Rosenzweig

**Affiliations:** 1 Department of Physics and Astronomy, University of California, Los Angeles, Los Angeles, California 90095, USA; 2 SLAC National Accelerator Laboratory, Menlo Park, California 94025, USA

## Abstract

There is urgent need to develop new acceleration techniques capable of exceeding gigaelectron-volt-per-metre (GeV m^−1^) gradients in order to enable future generations of both light sources and high-energy physics experiments. To address this need, short wavelength accelerators based on wakefields, where an intense relativistic electron beam radiates the demanded fields directly into the accelerator structure or medium, are currently under intense investigation. One such wakefield based accelerator, the dielectric wakefield accelerator, uses a dielectric lined-waveguide to support a wakefield used for acceleration. Here we show gradients of 1.347±0.020 GeV m^−1^ using a dielectric wakefield accelerator of 15 cm length, with sub-millimetre transverse aperture, by measuring changes of the kinetic state of relativistic electron beams. We follow this measurement by demonstrating accelerating gradients of 320±17 MeV m^−1^. Both measurements improve on previous measurements by and order of magnitude and show promise for dielectric wakefield accelerators as sources of high-energy electrons.

## Abstract (web summary)

Wakefield accelerators are a cheaper and compact alternative to conventional particle accelerators for high-energy physics and coherent x-ray sources. Here, the authors demonstrate a field gradient in excess of a gigaelectron-volt-per-metre using a terahertz-frequency wakefield supported by a dielectric lined-waveguide.

## Graphical abstract

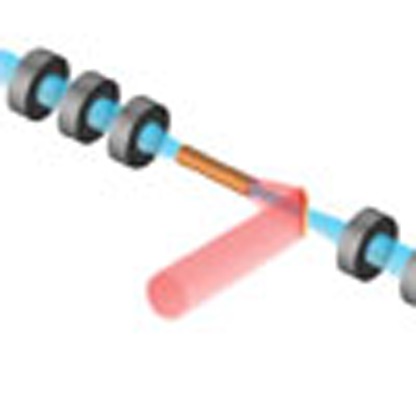

High-energy physics require ever increasing beam energies to reach the frontiers of new science. The result of this requirement is machines of ever increasing size and cost^1^. Acceleration gradients greater than gigaelectron-volt-per-metre (GeV m^−1^), exceeding those in current accelerators by an order of magnitude, may enable, for example, a sub-km TeV-class electron-positron collider^2^. Such a large instrument has similarities to the X-ray free-electron laser^3^ in the present need to construct lengthy >10 GeV (ref. 4) linear accelerators. As there is great demand for such sources^5^,^6^,^7^, ways of diminishing their size and cost may significantly enhance their already revolutionary impact.

In present frontier high-field linear accelerators (linacs) extremely large electromagnetic (EM) powers are needed to obtain the desired acceleration gradients. To mitigate this power demand acceleration at shorter EM wavelength, moving from the cm to the sub-mm terahertz (THz) regime, is favoured. However, between radio-frequency and infrared wavelengths there is a dearth of high-power sources. As such, development of new GeV m^−1^ acceleration techniques requires fundamental rethinking of the power source. In addition, traditional radio-frequency structures are limited by breakdown of the metallic cavity walls. Thus to support large amplitude longitudinal electric fields one must examine alternate power sources and media.

A wakefield based accelerator is a system in which an intense charged particle beam directly excites accelerating fields in a slow-wave, phase velocity *v*_ph_<*c*, structure or medium. This allows for the generation of large accelerating fields at wavelengths where no traditional power source exists. Here we experimentally investigate this promising means of generating and supporting GV m^−1^ fields using wakefield-excited dielectric tubes—the dielectric wakefield accelerator (DWA).

Wakefield schemes have been investigated intensively in the context of plasmas (plasma wakefield acceleration, PWFA), in which sustained acceleration of over 40 GeV m^−1^ in ∼1 m has been demonstrated^8^. More recently, high-efficiency acceleration has been demonstrated^9^, exceeding 50%, using a drive-witness bunch configuration. While the use of plasma permits extremely high-accelerating gradients, they also introduce difficulties such as instabilities, collisions and radiative processes that are not present in today's accelerators. Plasma wakefield schemes also encounter challenges in accelerating positively charged particles^10^. Investigation of optical-infrared accelerators based on dielectrics (dielectric laser accelerators, DLA)^11^ have shown deduction of >300 MV m^−1^ fields^12^,^13^ from an increase in injected beam energy spread. This recent experiment illustrates some difficulties of the DLA approach, which include breakdown-limiting fields at the ∼GV m^−1^ level, as well as production and transport of beams with unprecedented small spatial extent, given the sub-μm apertures and wavelengths found in optical-infrared DLA structures^14^. The THz-regime dielectric wakefield accelerator demonstrated here addresses the above mentioned challenges in addition to offering gradients of the same order of magnitude as THz frequency plasma-based PWFA and laser wakefield acceleration (LWFA) systems. Further, given the expanded operating wavelength used in THz DWAs, transport issues associated with DLA apertures are avoided. While DWA^15^ have shown structure breakdown accelerating field limits^16^ in excess of 5 GV m^−1^, acceleration gradients in excess of 69 MeV m^−1^ have not yet been demonstrated^17^,^18^,^19^.

In the following, we report here average drive bunch deceleration gradients of 1.347±0.020 GeV m^−1^ in a 15 cm long DWA, corresponding to a median energy change of over 202±3 MeV in a 20.35 GeV electron beam. We further show an accelerating gradient of 320±17 MeV m^−1^, with an associated wave-energy extraction efficiency of ∼80%, by dividing the electron bunch into a driver-witness pair.

## Results

### Experimental description

As an electron beam traverses the DWA, it couples to the structure EM modes via their longitudinal electric field, *E*_z_. Since the wakefields are excited in a dielectric material by ultra-relativistic particles the modes excited are classified as guided Cerenkov^20^ radiation. The peak *E*_*z*_ associated with such a coherent Cerenkov excitation process is estimated^20^,^21^ as *E*_*z*_∝*eN*_b_/*σ*^*2*^_*z*_, where *e* is the electron charge, *N*_b_ the number of beam electrons, and *σ*_*z*_ the root mean squared (r.m.s.) beam length. To obtain this scaling, we assume the transverse structure inner and outer radii (*a, b*) are proportional to *σ*_*z*_. Emission coherence is achieved through spatial localization of the radiating electrons, that is, using beams with longitudinal extent short compared to the mode wavelength *λ*, that is, *σ_z_*<*λ/2π*. For a given structure the mode frequencies are determined by the transverse boundary conditions^22^; thus large *E*_*z*_ is obtained when higher charge *eN*_b_ and small beam dimensions (*σ*_*z*_, *σ*_*x,y*_<<*a*) are used, where *σ*_*x,y*_ represents the r.m.s. beam size in the two transverse directions. Such beams are available at the Facility for Advanced Accelerator Experimental Tests (FACET) at SLAC National Laboratory, where the reported experiments were performed.

**Figure 1:**
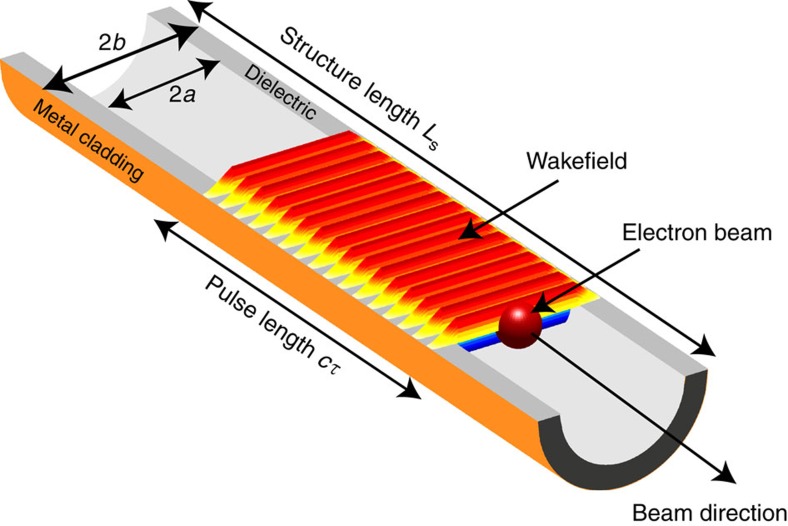
Graphical representation of dielectric wakefield accelerator. A cutaway view with the dielectric shown in grey and metal cladding in copper. The beam (dark red) travels along the structure in the vacuum region, leaving an idealized accelerating wakefield *E*_z_ shown in a colour intensity map (red to blue). The wakefield inside of the excited wave-train is shown as constant in magnitude as a function of distance behind the beam, consistent with theory and simulation assuming lossless media. This is in contrast to the experimental results in Fig. 4, which display dissipation effects.

The DWA structures utilized in these experiments are fabricated from SiO_2_ annular capillaries coated with an outer metal layer to form dielectric lined waveguides ranging in length *L*_s_ from 1 to 15 cm. The structure's cylindrical symmetry, as shown in Fig. 1, maximizes beam-radiation coupling. The hole in the dielectric gives a vacuum aperture that permits unobstructed near-axis beam passage.

The primary data presented in this work is the measured changes—through acceleration and deceleration—in the beam's kinetic state after interaction with the structure. Additionally, we characterize the beam's EM interaction with the DWA by examining properties of the coherent Cerenkov radiation (CCR) generated in the structure. This combination of two types of measurement, in concert with comparisons to theoretical models, yields strong insights into GeV m^−1^ acceleration in a DWA.

### Beam parameters

Relevant nominal FACET beam parameters are described as follows: a 20.35 GeV electron bunch consisting of ∼2 × 10^10^ particles, having r.m.s. bunch length *σ*_*z*_ adjustable between 20 and 50 μm (peak current of 18–45 kA, see Methods) is produced at a repetition rate of 1–10 Hz. The electron beam is then focused to ∼30 × 30 μm r.m.s. transverse size (*σ*_*x*,*y*_), with this small size maintained over tens of centimetre around the nominal interaction point. These beam parameters are optimum for exciting large wakefields in multi-centimetre length, THz DWA structures. This beam may also be split into two components^9^: a driver that excites the wakefield, and another witness that serves to measure the wakefield.

### Giga electronvolt-per-metre gradients

**Figure 2:**
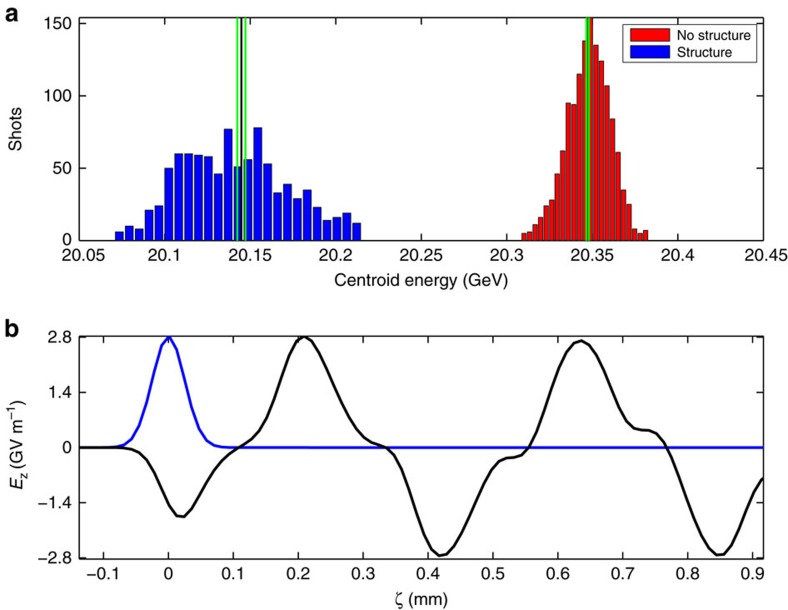
Measurement of large energy gradients in a dielectric wakefield accelerator. (**a**) Histogram of the electron beam centroid energy after traversing a 300 μm inner diameter, 400 μm outer diameter, 15 cm-long dielectric structure (blue, 1,385 measurements) and without the structure (red, 937 measurements). Black lines mark the mean and green lines the 95% confidence intervals. The distribution resulting after beam passage through the dielectric wakefield accelerator structure widens due to the parametric dependencies of the energy loss. The measured average energy loss is 202±3 MeV; the decelerating gradient is thus 1.347±0.020 GeV m^−1^. (**b**) Theoretical prediction of longitudinal wake for the case of (**a**), showing 2.8 GV m^−1^ peak fields (normalized beam current in blue).

We first discuss measurement of the electron beam's energy loss after traversal of a long, *L*_s_=15 cm SiO_2_ capillary (300 μm ID (2*a*), 400 μm OD (2*b*)). For this particular measurement a single 20.347±0.001 GeV beam consisting of 1.929 × 10^10^±0.003 × 10^10^ electrons with a bunch length *σ*_*z*_ of 25 μm and a peak current *I*_peak_=37 kA was used. The relevant data obtained consist of the beam centroid energy, repetitively measured in a charged particle spectrometer, for several thousand shots divided between those taken with and without the structure. The result of this process (see Methods), shown in Fig. 2, is the observation of an average beam energy change of 202±3 MeV, corresponding to an energy gradient of 1.347±0.020 GeV m^−1^. These measurements are consistent with theory and particle-in-cell (PIC) simulations, and correspond to a peak field behind the bunch of 2.8 GV m^−1^ (Fig. 2b); this is well over an order of magnitude more than the maximum used in current linacs^23^. In addition, the DWA structures operated for >28 contiguous hours, at 10 Hz rep rate (in excess of 100,000 pulses) and showed no signs of deterioration in performance.

### High-gradient acceleration

**Figure 3:**
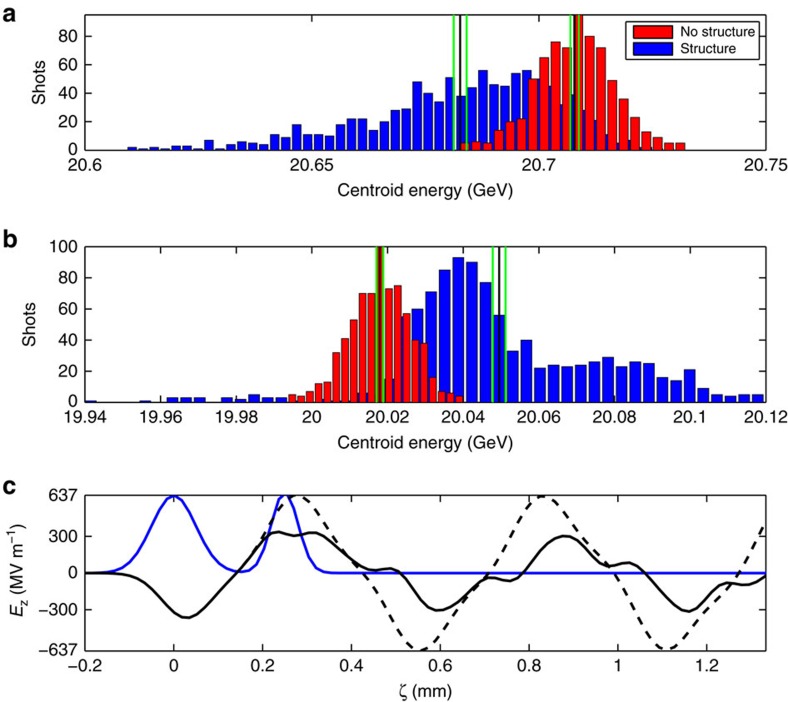
Measurement of accelerating gradients in a dielectric wakefield accelerator. Histogram of centroid energy of (**a**) the driver and (**b**) witness bunch. Blue and red indicate measurements made with (1,019 measurements) and without (722 measurements) the structure, respectively. A mean decelerating gradient of 252±14 MeV m^−1^ is observed in the driver; a mean accelerating gradient of 320±17 MeV m^−1^ is seen by the witness. (**c**) Theoretical prediction of wake from beam current (blue), with (black) and without (dashed) witness beam.

The second phase of the experiment measured the energy transferred from a decelerating driver bunch to an accelerating witness bunch. Here, a *L*_s_=10 cm long quartz structure of 400 μm ID (2*a*) and 530 μm OD (2*b*) was used, producing a measured fundamental (TM_01_) mode wavelength of 560 μm (see Methods). The longitudinal separation of the driver-witness pair was then optimized, using the measured TM_01_ wavelength, to 250 μm, placing the witness slightly ahead of the peak accelerating wakefield (*cf.* Fig. 3c). This placement is optimal to simultaneously obtain the highest gradient acceleration and largest efficiency. In this case, the driver and witness bunches contain 9.39 × 10^9^±0.06 × 10^9^ and 5.94 × 10^9^±0.05 × 10^9^ particles, with bunch lengths *σ*_*z*_ of 55 and 30 μm, respectively. The initial energy for the drive and witness bunches was 20.708±0.001 and 20.018±0.001 GeV, respectively. The transverse beam size for both the driver and witness beam was measured as *σ*_*x,y*_*=*30 μm r.m.s. We note that peak beam current and concomitant wake amplitude were sacrificed to produce this highly useful two-bunch structure, resulting in a drive bunch peak current of *I*_peak_=9.7 kA. The data set obtained, utilizing the same centroid energy change methods as the single bunch case described above, is summarized in Fig. 3. We observe a driver mean decelerating gradient equivalent to 252±14 MeV m^−1^ and a mean witness bunch accelerating gradient equivalent to 320±17 MeV m^−1^. This measurement represents the largest gradient ever observed via a witness bunch in a DWA by a factor of more than four. Further, from these average energy changes, it may be inferred that the efficiency of energy extraction from the wakefield by the witness beam is 80±4%—higher than demonstrated in recent PWFA experiments^2^. We note that for comparison with recent PWFA experiments^9^, we define the efficiency as the per cent of energy in the excited wakefield which is transferred to the witness bunch. A high-efficiency of beam loading also, per definition, diminishes the peak accelerating wake. Indeed, in the absence of the witness bunch the wake amplitude in this case is predicted by simulation to be 640 MV m^−1^, shown in Fig. 3c.

### Mode excitation in a DWA

**Figure 4:**
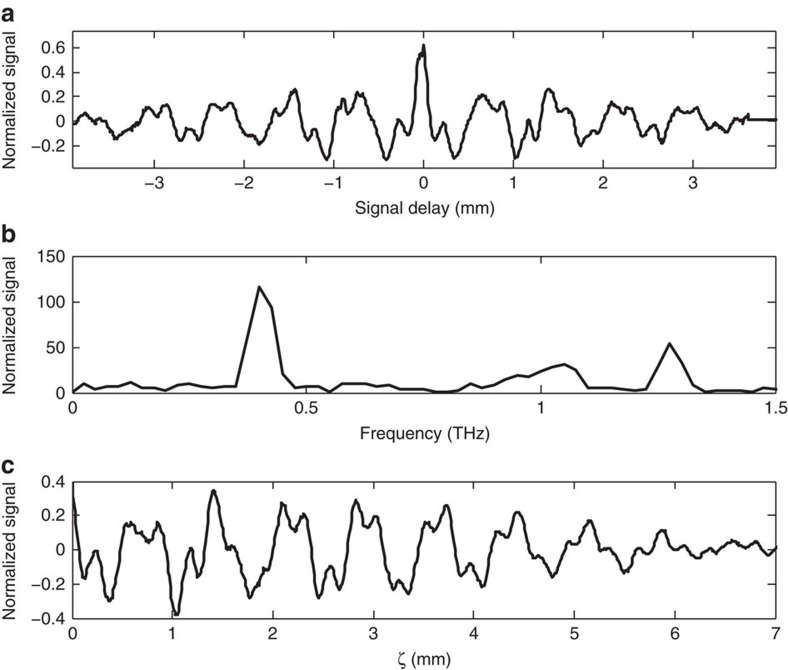
Modes excited in a dielectric wakefield accelerator. (**a**) Interferogram showing the autocorrelation of the coherent CCR generated by a single electron bunch in a 1 cm long dielectric wakefield accelerator structure. The central spike is due to prompt coherent diffraction radiation generated by structure exit and collection optics. (**b**) Spectral content of interferogram in (**a**), with the TM_01_ (422 GHz) and TM_02_ (1.27 THz) modes readily identifiable. Coherent diffraction radiation gives the broad peak near 1 THz. (**c**) Kramers–Kronig reconstruction of CCR (see Methods) in time domain from analysis of (**a**,**b**), plotted as the signal strength as a function of distance behind the driving beam, *ζ*=*z*−*ct*.

In addition to the measurement of changes in the beam's kinetic state after passage through the DWA structure, EM properties of the radiated modes in the structure are experimentally examined. This aspect of the measurement consists of obtaining the spectrum of the CCR radiation generated in the DWA through interferometric methods (see Methods). Such a study benchmarks the DWA's material and dimensional properties, and can yield information about the possible excitation of undesirable hybrid electric and magnetic (HEM) modes, which are excited when the beam and DWA are not collinear. Figure 4 shows an example of measurement of the CCR spectrum generated in the DWA, using a single electron bunch. For this case a *L*_s_=1 cm, 450 μm inner diameter (ID, 2*a*), 640 μm outer diameter (OD, 2*b*) SiO_2_ structure was employed. Excitation of the fundamental transverse magnetic TM_01_ (at 422 GHz) and next order TM_02_ (at 1.27 THz) modes is evident in the spectrum; the frequencies are in agreement with theoretical calculations. The first two HEM modes supported in this structure are expected near 350 and 585 GHz. The system used for positioning the DWA has a resolution of 2 μm or a maximum angle of 40 μrad from end-to-end in a 10 cm structure, so no excitation of the HEM modes was observed.

As a consequence of the methods used in the spectral measurement, time domain information is retrievable and reveals heretofore unobserved damping effect of the radiation generated in DWAs. Assuming a lossless dielectric, the radiation pulse length exiting the structures at group velocity *β*_g_*c*, where *β*_g_ is the group velocity of the mode and *c* the speed of light, should be proportional to *L*_s_, as *cτ*=*L*_s_(1−*β*_g_)/*β*_g_ (*cf*. Fig. 1). For this example the group velocity of the CCR in the structure is *β*_g_=0.39 and the TM_01_ bandwidth in the 1 cm structure is measured to be 14% FWHM, far exceeding the Fourier transform limit derived from the value associated with *cτ* of 1.8%. The observed CCR bandwidth from the *L*_s_=1 cm structure is an order of magnitude larger than that previously measured in similar structures with fields in the MV m^−1^ range excited by a 13 MeV electron beam^22^. When a 10 cm structure is used the bandwidth decreases to <5% FWHM; this is, however, even further from the lossless transform limit of 0.18% indicated by a CCR pulse length of *τ*=708 ps.

## Discussion

The observed deviation in bandwidth of the measured CCR spectra from theory is due to strong damping of the wakefield as it propagates, as is suggested by inspection of the autocorrelation (Fig. 4a). To quantify this observation in the time domain we apply a Kramers–Kronig analysis^24^. The signal reconstruction obtained (Fig. 4c) maps the wakefield-derived signal with 10 fs resolution (see Methods). This wakefield measurement method represents a powerful new approach in such techniques. The reconstruction yields a signal similar to the wakefield predicted and confirmed by both theory and simulations using fully EM PIC codes^25^ (not shown), but with amplitude damping superimposed. The wakefield modes calculated via direct solution of Maxwell's equations and PIC simulation were found to be in excellent agreement. However, the observed damping in experiment is much larger than that ascribable to dielectric or metallic losses under the conditions used in these experiments.

It should be emphasized that this observed damping does not impact the acceleration and deceleration studies presented in this work, as the time scale for these effects to assert themselves is >100 ps, while the driver-witness beams interact with a separation of less than a picosecond. Nevertheless, this damping is an unexpected feature—comparison with previous studies^24^ makes it clear that the observed damping is introduced by use of a very intense, short pulse electron beam. As such the basis of this effect can be uniquely studied at FACET, which will be further investigated in the future.

A measured gradient of 1.347±0.020 GeV m^−1^ was obtained in a THz frequency DWA and represent a significant step forward in advanced acceleration techniques in DWA. An acceleration gradient of 320±17 MeV m^−1^ was also shown, which is currently the highest acceleration gradient measured in a DWA. These results open the path towards exploration of critical issues in GeV m^−1^ DWA research. These issues include: extension of the interaction to longer structures and larger relative energy gains; excitation of HEM dipole modes in high-gradient structures and related transverse stability of driver and witness bunches in long DWAs^26^ (see Methods); experimental exploitation of CCR-derived THz; and clarification of the microscopic mechanism behind the wakefield dissipation and its implications for multi-bunch DWA operation, to name but a few. Beyond these next steps, there lay numerous avenues for development of new types of DWA structures based on dramatically different geometries, photonic mode confinement^27^ and novel materials. Such innovations may help pave the way to application of this promising GeV m^−1^ accelerator across a broad swath of science.

## Methods

### Metallic coatings

To form the DWA structure the SiO_2_ capillaries must be coated with a metal outer layer. The process of forming the coating involves first chemical cleaning the bare dielectric fibres. Following this, a 30 nm seed layer of aluminium is deposited onto the capillary using a vapour deposition system. The large electronegativity difference between the quartz and the aluminium ensures a strong covalent bond at the SiO_2_–Al surface. Next, a 500 nm layer of copper is vapour deposited over the aluminium, forming a metallic bond between the two metal layers. The structures are then transferred to an electroplating bath that is used to add copper to a thickness of ∼10 μm. Following the electroplating bath the structures are mounted into a holding chuck and a precision diamond saw is used to cut the structures to desired length. After cutting, the structure ends are polished using micrometre grain sandpaper and examined under a microscope for end surface finish (Supplementary Fig. 1).

### Beam-structure alignment

The electron beam is aligned to the DWA structures using a three-step process. To establish the electron beam's vector through the experimental region, with the DWA outside the beam path, the electron beam's position on diagnostic (optical transition radiation) screens is recorded by cameras located at the interaction point (IP), upstream and downstream of the experimental chamber. Electron beam operation is ceased and a green diode laser is steered to the electron beam trajectory utilizing the diagnostic screens. The laser is focused down to simulate the electron beam's transverse dimensions in the DWA interaction region. The DWA structure is then moved into position using the green laser as a guide, thus permitting the structure to be well aligned to the beam trajectory without the electron beam impinging on the structure and avoiding possible damage.

### Electron beam

The nominal beam energy is 20.35 GeV with an uncorrelated r.m.s. energy spread of *σ*_E_=298 MeV, or 1.1% of nominal energy. A typical beam consists of ∼2 × 10^10^ electrons, corresponding to 3.2 nC of total charge with repetition rate variable from 1 to 10 Hz. For this paper the current is defined assuming a Gaussian longitudinal beam form factor, *I*_peak_=*ceN*_e_/*σ*_*z*_. In calculation and simulation the measured parameters *N*_e_ and *σ*_*z*_ are used directly, leaving the definition of current as a matter of convention. The electron beam spot size at the IP is measured using optical transition radiation screen and wire scanner methods. The longitudinal extent of the driver and witness beams is measured using a transverse deflecting cavity^9^. For the experiments performed in this work the r.m.s. transverse sizes *σ*_x_ and *σ*_y_ were consistently near 30 × 30 μm, while the bunch length *σ*_*z*_ was varied from 25 to 50 μm. In a significant extension of FACET capabilities, the longitudinal beam profile was also strongly varied using momentum dispersion in tandem with selective collimation. This technique produced the option of creating a driver and witness pair; the methods utilized are described in detail in ref. 9.

### Coherent Cerenkov radiation measurements

The CCR that propagates downstream in the DWA tube after beam excitation exits the structure into impedance matched launching horn having a full opening angle of 19 degrees. The CCR is then propagated quasi-optically, first encountering an off-axis paraboloid (OAP) mirror, which serves to collimate the radiation. It is directed through a polymer-based vacuum window with optimum THz transmission characteristics and reflected into a scanning Michelson interferometer equipped with pyroelectric detectors. Scans having 10 fs temporal steps, and thus equivalent resolution, are then used to create an autocorrelation-based interferogram. A fast Fourier transform of the interferogram is performed and the spectral content of the CCR is obtained. This spectral amplitude information is further analysed to deduce the time-domain amplitude and phase via a Kramers–Kronig reconstruction analysis^24^, which reproduces the so-termed minimal phase consistent with the measured spectral intensity. This approach is robust for non-dispersed radiation signals, and has been shown previously to yield accurate results through benchmarking radiation profiles. Given the fixed relationship between the propagating transverse EM field measured at the interferometer and *E*_z_ within the DWA, this method gives a resulting form proportional to the time dependence of *E*_z_ for each mode at the exit of the DWA. While the amplitude relationship between the modes is not strictly preserved in this type of measurement, the method does robustly return the minimal phase, and thus the time domain wave form in this approximation, which is of central interest.

### Spectrometer (beam energy) measurements

The spectrometer consists of a pair of quadrupole magnets, used to transversely focus the beam after the IP, and a dipole magnet, which is used to disperse the beam's momentum distribution. The optics of the system are set so as to provide point-to-point imaging in the dispersive direction, as well as substantial magnification of position and angle in the non-dispersive direction. After the dipole the beam passes through a vacuum-air interface and subsequently impinges on a scintillating LANEX screen. The dispersed beam then passes through a pair of mirror finished silicon wafers that are 1.4 cm apart. Since the beam velocity exceeds the phase velocity of light in air, Cerenkov light is produced. This light is collected and imaged onto a 16-bit CMOS camera.

The spectrometer video imaging resolution evolved continuously due to improvements made during the two-year experimental period of the measurements here. The resolutions obtained for a static pixel size of 25 μm in various measurements ranged from 4.8 to 15.0 MeVper pixel. With the r.m.s. energy spread quoted above, the beam (full width, 6*σ*_E_) spans ∼100 pixels. Since we are interested in a measurement of the average, or centroid, of the beam momentum (and thus energy) distribution, we are performing a middle Riemann sum approximation to calculate the first moment of the beam's distribution function. The error in such an approximation is given by Error ≤*M*_2_(*f−g*)^3^/24*n*^2^, where (*f−g*) is the span of the integral approximated by the sum, *n* is the number of sub-intervals (pixels in this case) and *M*_2_ is the maximum of the absolute value of the second derivative of the function being integrated. For these calculations we assume the energy spread is well approximated by a Gaussian distribution, and thus *M*_2_=*σ*_E_^−2^, (*f−g*)=6*σ*_E_ and *n*=100. As a result of the single pixel resolution in the spectrometer the error in measuring the beam energy centroid is ≤0.2 MeV.

The first moment of the beam position distribution, as measured by a beam position monitor (BPM) in the spectrometer transport, with and without the DWA structure present, is taken as the centroid transverse position of the beam through the spectrometer system in the non-dispersive direction (Supplementary Fig. 2). A linear transport model is employed to determine the absolute offset and angle of the beam at the end of the DWA structure, that is due to this measured offset on the BPM when the structure is in the beam path, as compared with the nominal case without DWA present. This offset and angle is then propagated using an experimentally benchmarked beam-to-spectrometer optics model to account for any dipole induced transverse momentum that may appear as apparent longitudinal momentum (and thus energy) loss or gain in the spectrometer measurements.

### Histogram generation

To perform the energy loss measurements, spectrometer data is taken with and without the structure in the beam path. After alignment of the structure to the beam, the structure is inserted into and removed from the beam in periods of data acquisition lasting from 100 to 200 separate measurements. This approach reduces the possibility of fluctuations in beam energy affecting the result to an ignorable level. After data is collected each set of measurements has a region of interest (ROI) defined around the measured distribution peak to remove undue influence from background noise. In the case of the two-bunch measurements, the ROI is divided into two sub-ROIs, one for the driver and one for the witness, respectively (Supplementary Fig. 3). The measured particle count per pixel channel at a given momentum is combined with the measured spectrometer calibration and the first moment (centroid) of the beam distribution is recorded and binned in a histogram. Once all the data for a given measurement have been obtained, the mean of the centroid is calculated and the 95% confidence interval is formed.

Despite the mean and deviation being well quantified, fluctuations of the input beam parameters may still create changes in the distribution between data taken with the structure and data taken without the structure. These fluctuations convolve with the physical processes under study in these experiments and result in changes such as the wider distribution of data taken with the structure, shown in blue in Fig. 2, and the extreme events shown in the two-bunch data, shown in Fig. 3.

### Dipole modes

While excitation of HEM dipole modes supported in the DWA structures was not detected in the measured radiation spectra (*cf.* Fig. 4b), the effects of these modes were observed in the beam location as found by BPM inside of the spectrometer system. The excitation of these modes are due to asymmetries in the alignment of the beam to the structure axis, specifically they arise if the beam propagates off-axis. These modes generate fields that deflect the beam in a direction perpendicular to the nominal propagation direction, and thus change the beam transverse position downstream of the DWA.

The linear transport model that is used to account for offset, and angle in the measured centroid energy can be used to quantify the transverse momentum imparted to the beam as it traverses the structure. A plot of the beam position for the two-bunch experiment, shown in Supplementary Fig. 2, shows an r.m.s. spread in position of 12 μm without the structure and 78 μm with the structure. No offset in centroid is observed. Since the bunch separation of 250 μm represents a significant fraction of the excited wavelength we attribute the change in beam position entirely to motion of the witness beam. This results in an offset of the witness beam of 169 μm, which would indicate a transverse field of 4 MV m^−1^. In contrast, the high-gradient experiment shows a change in offset, at the BPM, of 864 μm, resulting in a transverse gradient of 13.7 MV m^−1^. Since the point at which the beam position is measured is dispersive the measured offset is not indicative of free space propagation. If the beam were allowed to propagate without a focusing system for containment the beam would move off by the width of the high-gradient structure, 300 μm, in 30 m.

### Energy spread

For the driver-witness case the energy spread of the driver beam is found to be virtually unchanged from 1.1% in measurements without the structure to 1.08% in measurements with the structure (Supplementary Fig. 4). The witness beam, however, is found to increase in energy spread, from 0.44% without the structure to 0.73% with the structure. The relative changes in energy spread are due to nonlinearities in the phase space prior to interaction with the structure, as well as non-trivial phase curvature of the wakefields used to transfer energy between the beams. An example of the latter effect is shown in Fig. 3c of the main paper in which different parts of the witness (or drive) beam can be seen to experience different gradients. This non-ideal growth in witness beam energy spread is due to the method of driver-witness beam generation. This method does not allow simultaneous control of beam current profile, driver-witness separation and shaping for ideal energy spread conservation. As the former two are necessary for proper excitation and acceleration of the witness beam, the third is constrained and thus energy spread growth is observed.

### Beam divergence

The initial divergence of the driver and witness beams, in the plane perpendicular to the dispersion induced by the spectrometer, is calculated to be 8.3 × 10^−5^ rad. After interaction with the structure the drive beam divergence is calculated to grow to 1.7 × 10^−4^±1.7 × 10^−5^ rad, and the witness beam divergence increases to 1.35 × 10^−4^±1.2 × 10^−5^ rad. This growth is due to the aforementioned excitation of dipole modes in the structure. The excess growth of the drive beam compared with the witness beam is a consequence of the longer bunch length of the drive beam resulting in a larger sampling of the curvature of the gradients in the structure. This larger variance in longitudinal field results in a larger variance in transverse fields, and thus larger divergence changes, a consequence of the Panofsky–Wenzel theorem. This calculation is made making use of the fact that no change in the beam size is observed at the exit of the structure, so that all changes in beam size (Supplementary Fig. 5) can be attributed to a change in the beam's divergence through transverse momentum imparted by the wakefields.

### Data availability

All data supporting the findings of this study are available from the authors.

## Additional information

**How to cite this article:** O'Shea, B. D. *et al*. Observation of acceleration and deceleration in gigaelectron-volt-per-metre gradient dielectric wakefield accelerators. *Nat. Commun.* 7:12763 doi: 10.1038/ncomms12763 (2016).

## Supplementary Material

Supplementary Information Supplementary Figures 1-5
