# Supplementary material for: Observation of acceleration and deceleration in gigaelectron-volt-per-metre gradient dielectric wakefield accelerators
Source: Nat Commun. 2016 Sep 14;7:12763. doi: 10.1038/ncomms12763 (PMC5027279; doi:10.1038/ncomms12763)
Supplement: Supplementary Information — Supplementary Figures 1-5 [file ncomms12763-s1.pdf]

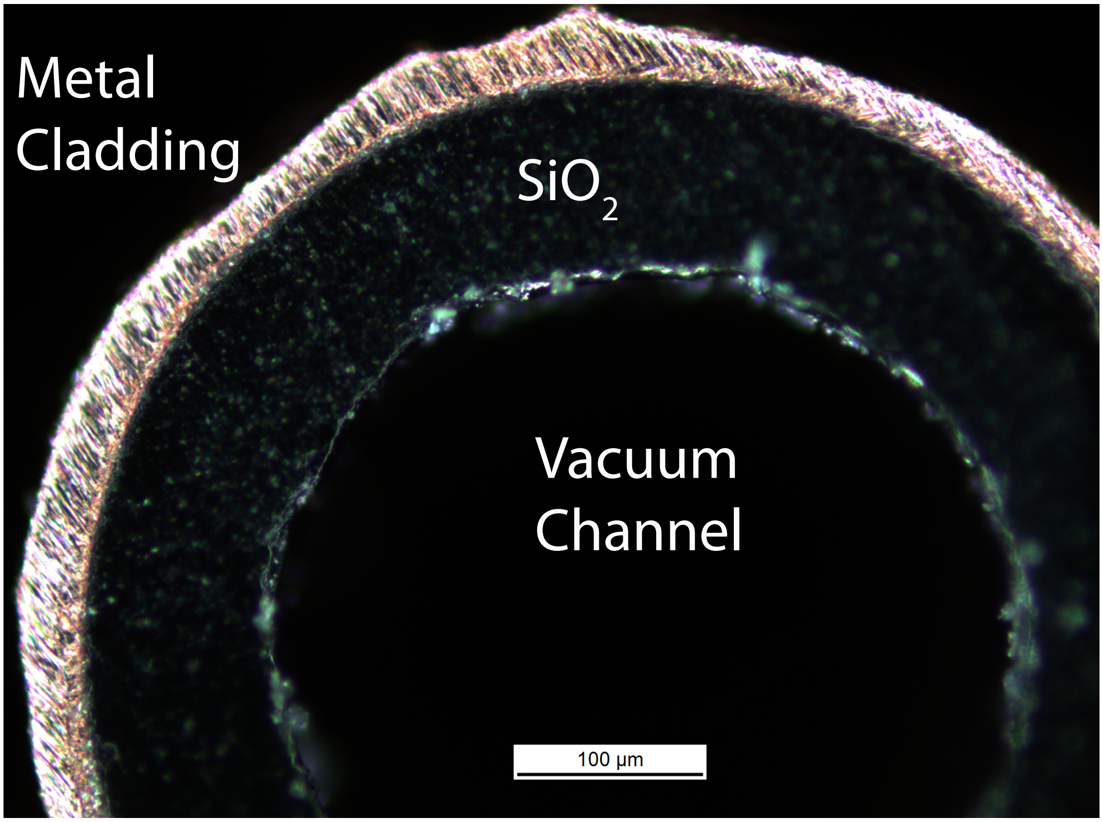

Supplementary Figure 1 **Structure photo** A high-resolution image of the end of a 400 μm inner diameter, 530 μm outer diameter structure. To ensure proper waveguide fabrication, images are taken of the structures prior to their installation in the experimental apparatus at FACET. Additionally, the vacuum apertures are checked by visual inspection and laser transmission techniques to ensure that they are not blocked by debris during the fabrication process.

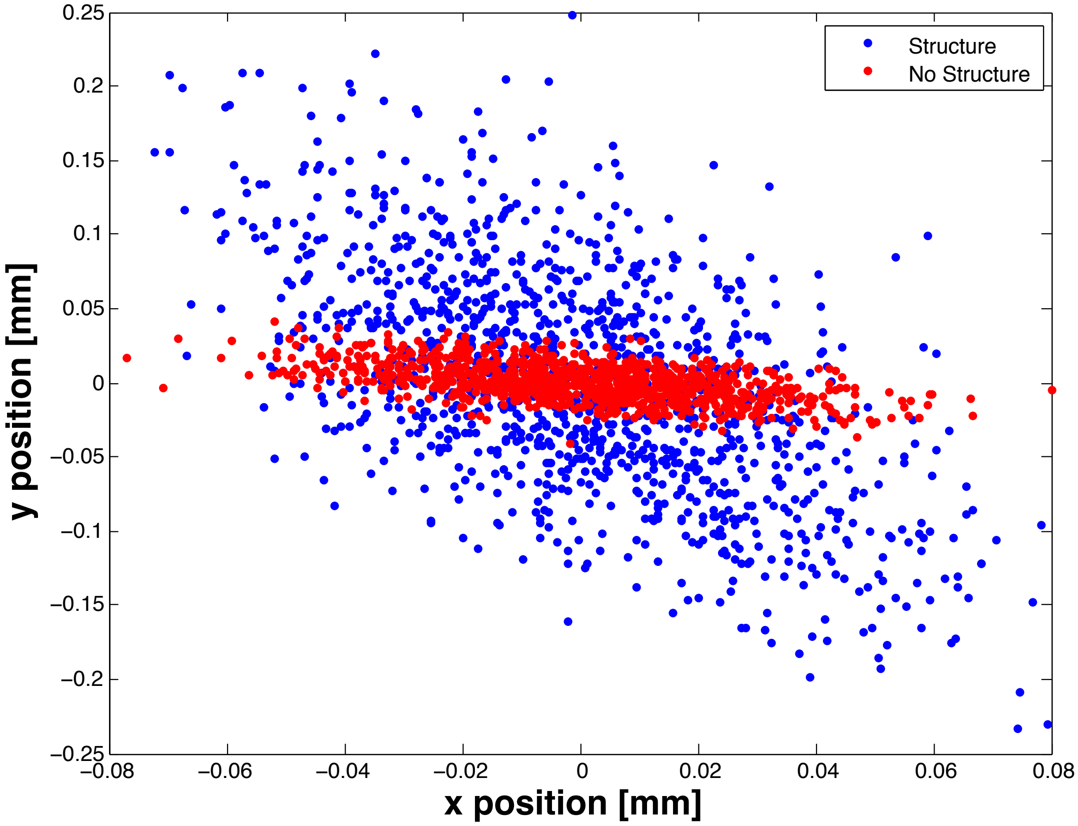

Supplementary Figure 2 **<u>Beam position data after structure</u>**
Beam position downstream of the structure location with (blue) and without (red) the structure in the beam path, as measured using a beam position monitor in a dispersive section of the spectrometer. Here we show observed coupling to transverse modes, via a relative shift in the position of the beam. The structure parameters do not allow for an appreciable shift in beam position over the length of the interaction. As such, the measured positions are due to shifts in transverse momentum (angle) and absolute beam energy.

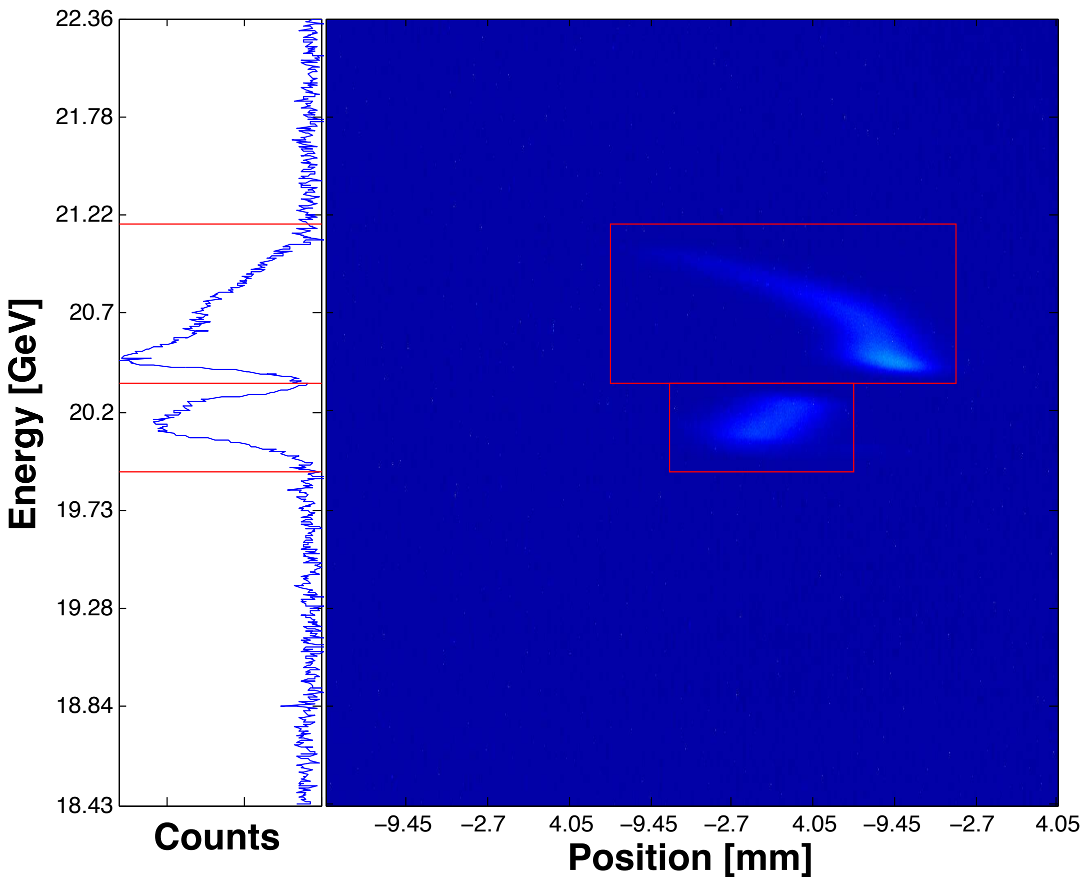

Supplementary Figure 3 **Spectrometer image** An example of the driver (top) and witness (bottom) beams after interaction with the dielectric wakefield accelerator, after transport through the spectrometer. The red boxes indicate the region of interest used for calculation of the energy and position of the beam. On the left the relative charge (represented by the counts in the pixels of the CCD camera) at each energy is shown. The limits for the region of interest are again shown in red.

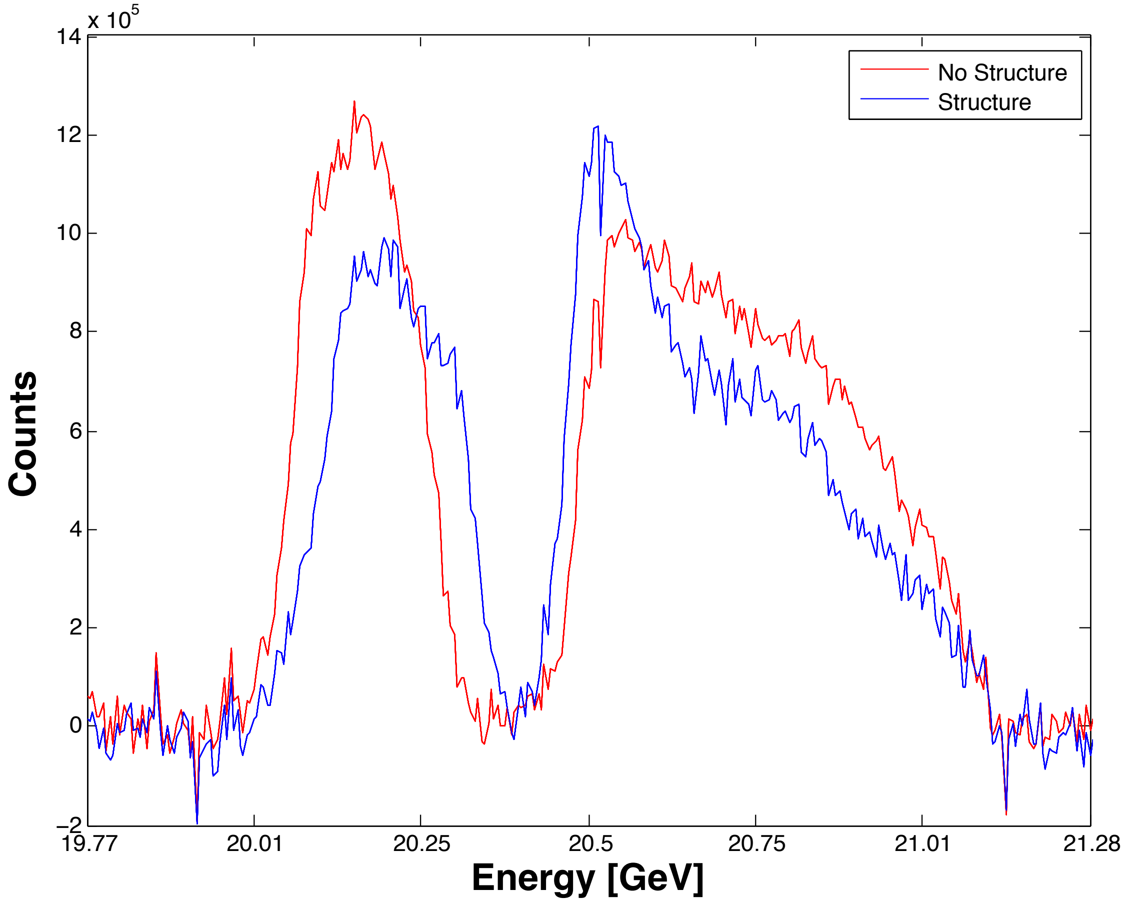

Supplementary Figure 4 **Driver and witness energy spectra.** The energy spectra for the case of interaction with the dielectric wakfield accelerator (blue) and no structure (red). The drive beam is on the right, while the witness beam is on the left. The energy spread for the drive beam is seen to be approximately conserved, at 1.1% prior to deceleration and 1.08% after. The energy spread of the witness beam is seen to increase from 0.44% prior to acceleration to 0.73% after acceleration.

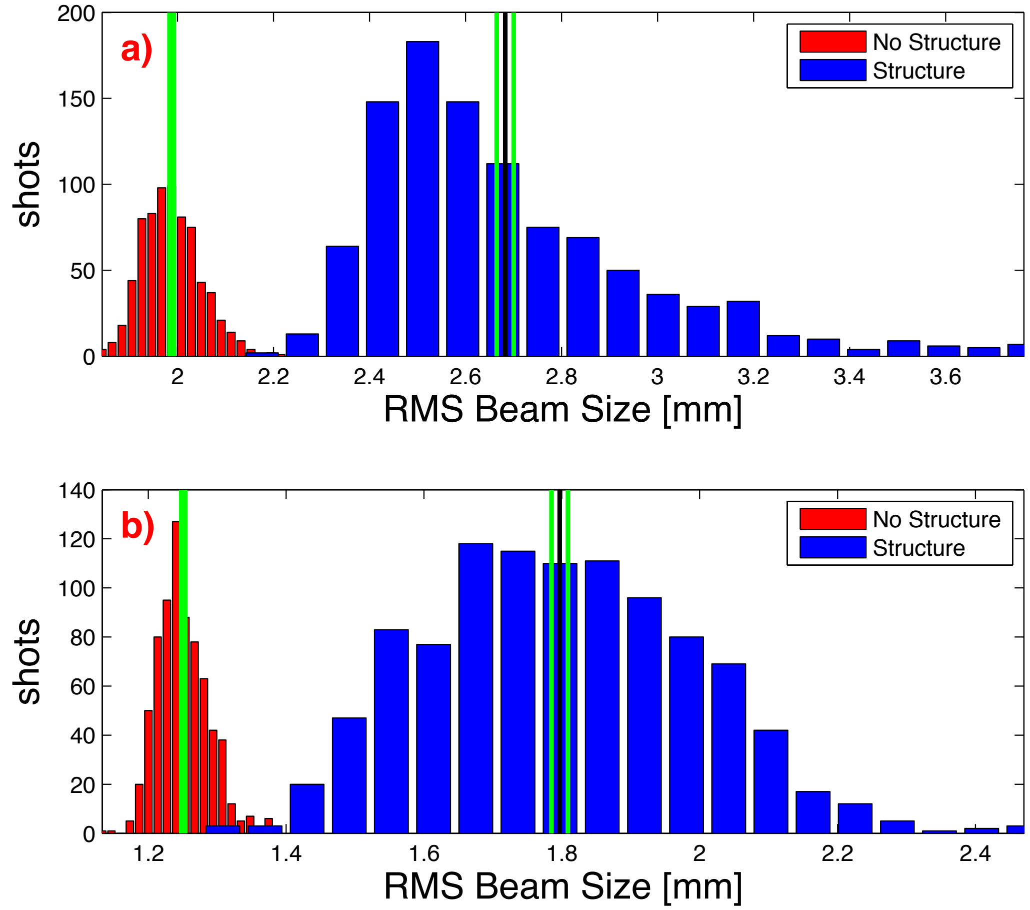

Supplementary Figure 5 **<u>Drive and witness beam sizes</u>** a) The beam sizes of the drive and b) witness beams with (blue) and without (red) interaction with the structure as measured in the spectrometer. The increase in beam size is commensurate with an increase of the driver and witness beam's divergence of 100% and 61% respectively. The black lines represent the mean and the green lines the 95% confidence interval. For the no structure case the confidence interval is small enough to obscure the black line that is the mean.
